# CD117 (KIT) in canine soft tissue sarcoma: an immunohistochemical and c-kit gene mutation assessment

**DOI:** 10.3389/fvets.2025.1572923

**Published:** 2025-04-09

**Authors:** Silvia Dell’Aere, Valentina Balbi, Damiano Stefanello, Giancarlo Avallone, Gabriele Ghisleni, Stefano Perfetto, Roberta Ferrari, Luigi Auletta, Elisa Maria Gariboldi, Alessandra Ubiali, Caterina Romanello, Alessandra Verdi, Paola Roccabianca

**Affiliations:** ^1^Department of Veterinary Medicine and Animal Sciences—DIVAS, University of Milano, Lodi, Italy; ^2^Department of Veterinary Medical Science—DIMEVET, Bologna University, Bologna, Italy; ^3^BiEsseA Laboratorio Analisi Veterinarie, an Antech Company, Mars Petcare, Science & Diagnostics, Milan, Italy

**Keywords:** canine, CD117, KIT, soft tissue sarcoma, therapeutical target, tyrosine kinase

## Abstract

**Introduction:**

Canine soft tissue sarcomas (STSs) are locally aggressive mesenchymal tumors with variable recurrence rates, and often, their therapy is limited to surgical excision. CD117 (KIT) is a tyrosine kinase receptor involved in cell growth and cancer development. c-kit proto-oncogene mutations have been reported to be associated with prognosis and therapy response in human and canine cancers. However, CD117 expression and c-kit mutations have rarely been investigated in canine STSs. This study aims to assess CD117 expression and c-kit mutations in different canine STSs.

**Methods:**

Spontaneous STSs were surgically removed, fixed, routinely processed, and stained for histological and anti-CD117 immunohistochemical analyses. Staining intensity and percentage of positivity were scored. Cases with intense CD117 expression in more than 50% of cells were analyzed for the presence of mutations in exons 8, 9, or 11 of the c-kit proto-oncogene.

**Results:**

Overall, 115 canine STSs were collected. Among them, CD117 was expressed in 43 STSs, with diffuse cytoplasmic staining of variable intensity. CD117 was expressed in 16 out of 27 perivascular wall tumors, 12 of 13 sarcomas of fibroblastic origin, 6 of 6 rhabdomyosarcomas, 7 of 46 liposarcomas, and 2 of 3 nerve sheath tumors. Leiomyosarcomas (20 of 20) did not show CD117 expression. Mutations were investigated in 22 cases, all of which returned negative results.

**Discussion:**

In summary, canine STSs variably expressed CD117, which suggests that tyrosine kinase inhibitors may represent a promising targeted therapy for selected canine STSs histotypes.

## Introduction

Canine soft tissue sarcomas (STSs) are a heterogeneous group of malignant mesenchymal neoplasms, accounting for 8–15% of all canine cutaneous and subcutaneous tumors (1). They show a variable biological behavior, from locally aggressive, with a low tendency to metastasize, to highly aggressive and destructive neoplasms (1–5). Thus, several studies have recommended a more specific approach to each tumor type to gain a better understanding of their behavior and response to therapy (6, 7).

Currently, the primary treatment for STSs is surgical removal (if feasible) (1, 3, 8, 9) in combination with radiotherapy, metronomic chemotherapy, or electrochemotherapy (10), particularly when the margins are infiltrated. While therapies aimed at controlling the recurrence of STSs have shown effectiveness to some extent, their efficacy in preventing metastasis is limited. Furthermore, prognosis tends to be worse for tumors exceeding 5 cm, and the response to therapy varies depending on specific histotypes (11). Despite the promising results of radiation therapy and metronomic chemotherapy following excision, new strategies, such as the expansion of anticancer drug availability to manage unresectable canine STSs and to improve their clinical outcomes, are being explored (7, 11–13). Therefore, targeted adjuvant therapies are being investigated to improve the management of inoperable tumors, control metastases, and reduce local recurrences.

CD117 (KIT) is a transmembrane receptor tyrosine kinase (RTK) that plays a role in the intracellular signal transduction pathways involved in cell growth, survival, and angiogenesis (14). The role of CD117 expression has been investigated in human medicine across various tumors (15–21), and its dysregulation has been shown to have implications in the development of gastrointestinal stromal tumors (GISTs), melanomas, acute myeloid leukemias (AMLs), and seminomas (16, 22). In veterinary medicine, CD117 expression has been investigated extensively in canine GISTs (23) and canine mast cell tumors (MCTs) (24–27). Sparse information is available about CD117 expression in other tumor types in canines. Studies have primarily focused on the differential diagnosis of GISTs and leiomyosarcomas (28, 29) and carcinomas (30–35), but studies on canine STSs are scarce (23, 35, 36).

Mutations in the c-kit proto-oncogene, especially in exons 8, 9, 11, and 17, lead to CD117 dysregulation and oncogenesis (16, 37). These mutations have been extensively investigated in human medicine due to their implications as prognostic factors related to survival time and as response to targeted therapy (21, 22). In veterinary medicine, c-kit mutations have been studied only in canine MCTs (24), where they are associated with increased recurrence rates (27) but do not predict the response to tyrosine kinase inhibitor (TKI) treatment (38), and in canine GISTs (39), where the administration of TKIs has proved successful despite the absence of c-kit gene mutations (39). In addition, c-kit gene mutations have been sporadically reported in dogs with hemangiosarcomas (40), melanomas (41), STSs (41), and osteosarcomas (41).

CD117 represents a good target for TKIs, a class of molecules acting as specific antineoplastic drugs that are effective in controlling cancers in humans (42) and, more recently, in companion animals (43).

Given the scarcity of available data, the present study aims to assess the protein expression of CD117 and mutations in different canine STS histotypes and investigate the potential of TKI targeting for improving therapeutical management in selected canine STSs.

## Materials

### Ethics

This study was approved by Organo Preposto al Benessere Animale (OPBA), the Institutional Animal Welfare Organization of the University of Milano, Italy (protocol no.: 60-2022).

The experiments conducted in this study involved privately owned dogs that spontaneously developed STSs. Internationally established and recognized high standards (“best practice”) of veterinary clinical care for the individual patient were always followed. Written informed consent was obtained from dog owners for all diagnostic and treatment procedures as well as for the use of data for scientific purposes.

### Case selection

Tissue samples included in the study were derived from either incisional biopsy or curative intent surgery of spontaneous canine STSs performed between 2002 and 2023 and stored in the archives of the Department of Veterinary Medicine and Animal Sciences (DIVAS), University of Milano, Italy; Department of Veterinary Medicine (DIMEVET), University of Bologna, Italy; and BiEsseA-Scil Laboratorio Analisi Veterinarie, Milano, Italy.

For all samples, information on dog signalment, tumor site, and diagnosis was collected.

### Histopathological analysis

Histological slides were reviewed by four pathologists, among whom two pathologists (GA and PR) were certified by the European College of Veterinary Pathology (ECVP) board, one ECVP resident (SD), and one doctor of veterinary medicine (DVM, GG). A histopathology analysis was performed through visual examination of 4-μm sections of formalin-fixed paraffin-embedded (FFPE) tissues, which were routinely processed and stained with hematoxylin and eosin (H&E). Tumors were histologically classified, subtyped, and graded according to the most recent classification (6). Definitive tumor diagnoses were confirmed using selected immunohistochemical (IHC) markers when only morphological features were insufficient to determine the histotype with certainty, as previously performed (44, 45). Diagnoses of doubtful cases of malignant nerve sheath tumors (MNSTs) were confirmed by the positive expression of nerve growth factor receptor (NGFR), while the diagnoses of rhabdomyosarcomas (RMSs) were confirmed by the positive expression of desmin.

Besides diagnosis, morphological features such as the percentage of necrosis and mitotic count were assessed for grade assignment. In addition, histological sections were investigated for necrosis by visually estimating the percentage of the section that was necrotic and categorizing it as absent, less than 50%, or more than 50% (6, 46). Mitotic counting was carried out using an Olympus BX51 microscope with a 40x objective and a 10x FN22 ocular eyepiece, counting 10 non-overlapping fields of the tumor, which corresponded to an area of 2.37 mm^2^, the standard area to be counted for grading (47–49). Fields were selected in less differentiated and more cellular areas that showed no to minimal microhemorrhage or edema and lacked necrosis. Histological grading was performed in accordance with the Surgical Pathology of Tumors of Domestic Animals (6).

### Immunohistochemical analysis

CD117 expression was analyzed on 4-μm paraffin sections by mounting them on poly-L-lysine-coated slides and staining them with an anti-CD117 primary antibody (Dako, Agilent Technologies, Glostrup, Denmark), whose cross-reactivity with canine tissues had been demonstrated previously (50–52).

Briefly, the sections were subjected to combined deparaffinization, hydration, and antigen retrieval in a pH 9.0 working solution (Buffer H, Epredia, Breda, Netherlands) that was preheated at 97°C in a water bath for 20 min, followed by the quenching of endogenous peroxidase activity with 0.3% hydrogen peroxide and incubation with anti-CD117 primary antibody at a 1:400 ratio for 1 h at room temperature. After 30 min of incubation with a biotinylated secondary antibody (goat anti-rabbit, Vector, CA, United States) at room temperature, detection was performed using the avidin–biotin enzyme complex (ABC Kit, Vectastain, Vector, CA, United States) for 30 min. The reaction was developed using a diaminobenzidine substrate kit (ImmPACT DAB Kit, Vector, CA, United States). Then, sections were counterstained with Mayer’s hematoxylin (Diapath, Bergamo, Italy), dehydrated, and mounted with Micromount (Diapath, Bergamo, Italy). A positive control from Patnaik grade 2, Kiupel low grade mast cell tumor with intense membrane and cytoplasmic CD117 positivity was included in each run. Negative controls consisted of primary antibody replacement with an isotype-matched irrelevant antibody (Rabbit Anti-von Willebrand Factor, Dako, Agilent Technologies, Glostrup, Denmark) or omission of the primary antibody.

The staining was semi-quantitatively evaluated by visually estimating the percentage of positive neoplastic cells (−, 0%; −/+, <5%; 1+, 6–25%; 2+, 26–50%; 3+, 51–75%; 4+, >75%). Cases expressing CD117 in more than 50% of neoplastic cells were considered to have high CD117 expression.

IHC staining included the estimation of staining intensity (1, weak; 2, intermediate; 3, strong) and its cellular localization (membrane, cytoplasm, and nucleus).

### Mutations

Polymerase chain reaction (PCR) amplification of exons 8, 9, and 11 of c-kit was performed on samples from dogs with high CD117 expression (i.e., in more than 50% of neoplastic cells) with strong intensity to identify mutations in the nucleotide sequence of the proto-oncogene. From selected FFPE tumor samples, 10-μm-thick sections were cut and placed in microcentrifuge tubes. Genomic DNA was extracted using the Maxwell RSC DNA FFPE Kit (Promega, Madison, WI) following the manufacturer’s instructions. The concentration of DNA was evaluated following a fluorometric procedure using a Quantus Fluorometer (Promega, Madison, WI). PCR amplification of all exons of the c-kit proto-oncogene was performed using previously described primer sets and cycling conditions (38, 53).

PCR products were visualized on a QIAxcel Capillary Electrophoresis System (Qiagen, Venlo, The Netherlands), and an aliquot of samples was also analyzed using polyacrylamide gel electrophoresis (PAGE) with a 6% polyacrylamide gel.

### CD117 expression associations

The association between the STS histotype, histological grade, and CD117 expression in STSs was assessed using Pearson’s χ2 test, and the data were analyzed using JASP (version 0.18.3) software. All *p*-values were from two-sided tests, and a *p*-value of <0.05 was considered statistically significant.

## Results

### Cases

A total of 115 cases were included in the study. Information on the dogs’ signalment and clinical presentation is presented in detail in Supplementary Table 1 and summarized in Table 1. Of the 115 dogs included, 68 were male, of which 9 were castrated, and 44 were female, of which 18 were spayed; for 3 dogs, information on sex and reproductive status was not available. The age range of these dogs ranged from 1 to 16 years, with a mean age of 9.9 years. In addition, 69 dogs were pure breeds, with Labrador Retriever (17), Golden Retriever (7), German Sheperd (4), and Rottweiler (4) being the most represented breeds; 41 dogs were crossbreeds, and information about the breed was not available for 5 dogs.

**Table 1:** Summary of selected soft tissue sarcoma and sex, age, and breed of the dogs included in the study.

Histotype	Total	Female dogs (total)	Neutered females	Male dogs (total)	Neutered males	Unknown sex	Age range (years)	Mean age (years)	Pure breed	Crossbreed	Unknown breed
Liposarcomas	46	10	2	35	3	1	4–16	10	31	12	3
Perivascular wall tumors	27	9	5	18	5	0	2–15	11	16	11	0
Leiomyosarcomas	20	10	5	10	1	0	4–15	10	9	11	0
Fibroblastic sarcomas (fibrosarcomas, myxosarcomas)	13	10	4	2	0	1	1–16	7.5	8	5	0
Rhabdomyosarcomas	6	3	0	2	0	1	1–11	4	4	0	2
Nerve sheath tumors	3	2	2	1	0	0	8–10	9	1	2	0
Total	115	44	18	68	9	3	1–16	10	69	41	5

The site of STS development was cutaneous/subcutaneous in 101 out of 115 dogs; of these, 40 were localized in the limbs (22 in the hind limb and 18 in the forelimb), 24 in the trunk, 13 in the head and neck region, 10 in the axilla, 8 in the perineum, 4 in the inguinal region, and 1 case each on back and tail. Among the total of 115 dogs, STS was intracavitary in 8 dogs, with localization in retroperitoneum in 4 dogs, pelvic cavity in 3 dogs, and mesentery in 1dog, and the site of STS development was unknown in 6 dogs.

### Histopathology

A summary of tumor subtypes and grades is presented in Table 2, and further details are included in Supplementary Table 1.

**Table 2:** Histotype and histological grade of canine soft tissue sarcomas included in the study.

Histotype	Grade I	Grade II	Grade III	Total
Liposarcomas	18	23	5	46
Perivascular wall tumors	10	13	4	27
Leiomyosarcomas	11	6	3	20
Fibroblastic sarcomas (fibrosarcomas, myxosarcomas)	9	4	0	13
Rhabdomyosarcomas	2	1	3	6
Nerve sheath tumors	3	0	0	3
Total	53	47	15	115

Tumors included 46 liposarcomas, subclassified into pleomorphic (24), well-differentiated (15), myxoid (4), and dedifferentiated (3); 27 perivascular wall tumors (PWTs); 20 leiomyosarcomas; 13 sarcomas of fibroblastic origin, subclassified into fibrosarcomas (7) and myxosarcomas (6); 6 RMSs; and 3 MNSTs.

Of the 115 tumors, 53 were grade I, 47 grade II, and 15 grade III.

### Immunohistochemical expression of c-kit

A summary of IHC staining results are presented in Table 3, and further details are included in Supplementary Table 1. CD117 expression was detected in 43 out of 115 tumors. All myxosarcomas and RMSs expressed CD117.

**Table 3:** Immunohistochemical expression of CD117 in canine soft tissue sarcomas included in the study.

			Cases with positive neoplastic cells					Staining intensity		
Tumor type	No. of cases	No. of positive cases (%)	-	1+	2+	3+	4+	Weak	Intermediate	Strong
Liposarcomas	46	7 (15%)	39	0	0	0	7	0	2	5
Perivascular wall tumors	27	16 (59%)	11	4	1	5	6	3	9	4
Leiomyosarcomas	20	0 (0%)	20	0	0	0	0	0	0	0
Sarcomas of fibroblastic origin (fibrosarcomas, myxosarcomas)	13	12 (92%)	1	0	2	3	7	0	5 †	7
Rhabdomyosarcomas	6	6 (100%)	0	2	0	2	2	1 ‡	2	3 §
Nerve sheath tumors	3	2 (67%)	1	1	0	0	1	1	0	1
Total	115	43	72	7	3	10	23	5	18	20

Symbols: -, <5% positive neoplastic cells; 1+, 6–25% positive neoplastic cells; 2+, 26–50% positive neoplastic cells; 3+, 51–75% positive neoplastic cells; 4+, >75% positive neoplastic cells; †, two cases showed variable staining intensity, from intermediate to strong; ‡, one case showed variable staining intensity, from weak to strong; §, one case showed variable staining intensity, from intermediate to strong.

Of the 46 liposarcomas assessed, 7 expressed CD117, of which 2 were grade I, 2 were grade II, and 3 were grade III. Among the positive liposarcomas, 2 were well-differentiated, 2 were pleomorphic, 2 were myxoid, and 1 was dedifferentiated.

**Figure 1:**
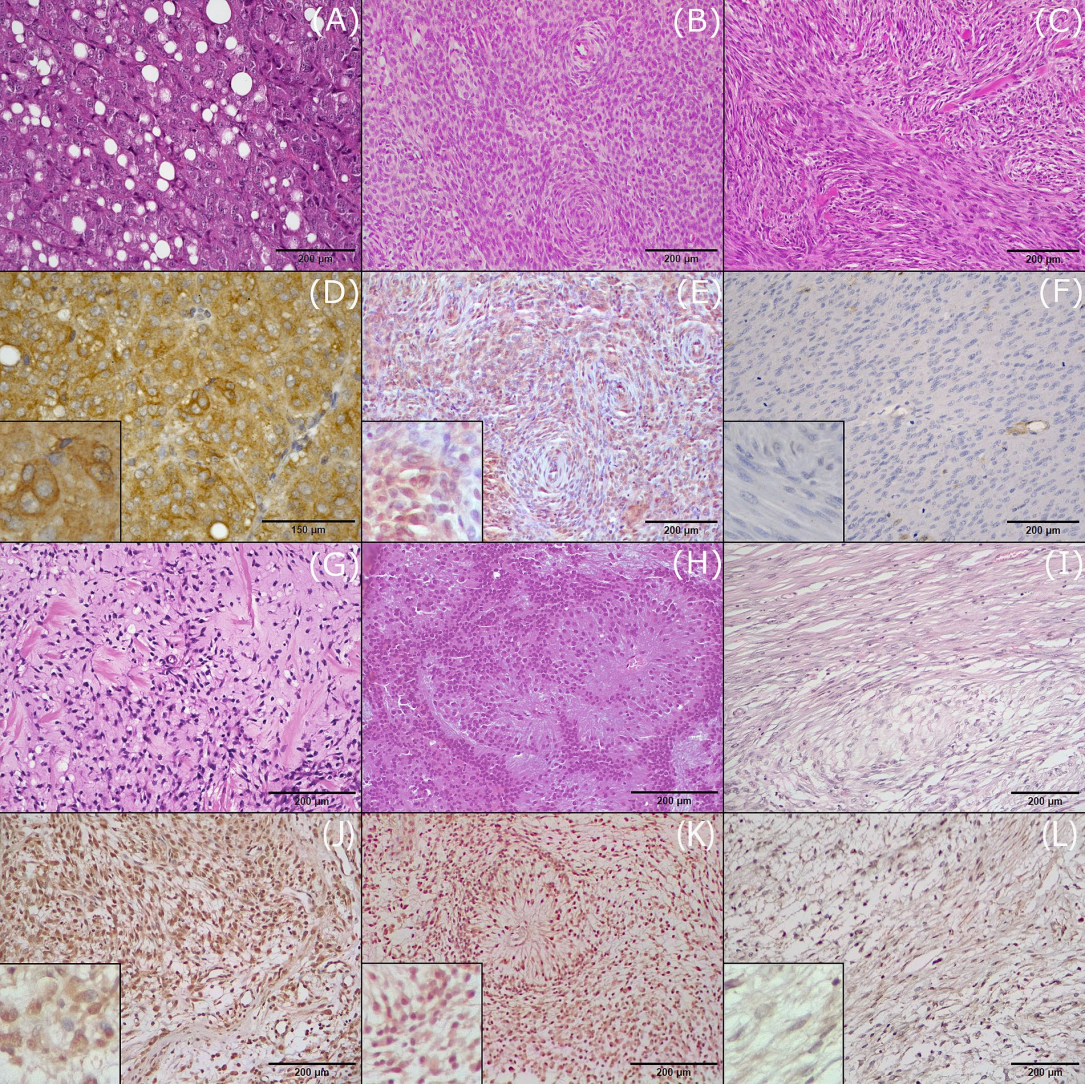
Hematoxylin and eosin (H&E) staining and immunohistochemical (IHC) expression of CD117 in the investigated histotypes of canine soft tissue sarcomas. **(A)** Liposarcoma, grade II, H&E. **(B)** Perivascular wall tumor, grade III, H&E. **(C)** Leiomyosarcoma, grade II, H&E. **(D)** Liposarcoma, grade II, IHC. CD117 is expressed in more than 75% of neoplastic cells (score 4+) with strong intensity (score 3). **(E)** Perivascular wall tumor, IHC. CD117 expression in 50–75% of neoplastic cells (score 3+) with weak to intermediate intensity (score 1). **(F)** Leiomyosarcoma, grade II, IHC. Negative expression of CD117. **(G)** Fibrosarcoma, grade I, H&E. **(H)** Rhabdomyosarcoma, grade II, H&E. **(I)** Malignant nerve sheath tumor, H&E. **(J)** Fibrosarcoma, grade I, IHC. CD117 is expressed in more than75% of neoplastic cells (score 4+) with strong intensity (score 3). **(K)** Rhabdomyosarcoma, grade II, IHC. CD117 expression in 50–75% of neoplastic cells (score 3+) with variable intensity from weak to strong (score 2). **(L)** Malignant nerve sheath tumor, CD117 expression in 5–25% of neoplastic cells (score 1+) with weak intensity (score 1).

In all positive liposarcomas, CD117 expression was cytoplasmic and was observed in more than 50% of neoplastic cells, and staining intensity ranged from intermediate to strong (Figures 1A,D).

Of the 27 PWTs assessed, 16 expressed CD117, among which 8 were grade I, 5 were grade II, and 3 were grade III. Among these 16 cases, CD117 expression was high in 11, with 4 being grade I, 4 being grade II, and 3 being grade III. Staining was cytoplasmic in all but one case, which showed nuclear expression of CD117. Staining intensity was strong in 4 out of 16 cases, intermediate in 9 cases, and weak in 3 cases (Figures 1B,E).

All 20 leiomyosarcomas were negative for CD117 expression (Figures 1C,F). In addition, there were 13 sarcomas of fibroblastic origin (fibrosarcoma and myxosarcoma) mentioned in this study, among which 12 expressed CD117 (Figures 1G,J). CD117 expression was assessed in seven fibrosarcomas: three grade I and four grade II. Of these seven tumors, four (two grade I and two grade II) tumors expressed CD117 in more than 50% of neoplastic cells; two tumors expressed CD117 in less than 50% of cells; and one tumor was negative. CD117 staining was cytoplasmic in all positive cases, with strong intensity in three cases (two grade I and one grade II), intermediate in two cases (one each of grade I and grade II), and variable (intermediate to strong) in one case (grade II).

Furthermore, six myxosarcomas were assessed. All these were grade I and expressed cytoplasmic CD117 in more than 50% of neoplastic cells, with intermediate to strong intensity.

Six RMSs were included in this study, consisting of one embryonic RMS of the rhabdomyoblastic variant, two pleomorphic RMSs, and three cases with an undefined subtype. All RMSs expressed CD117. High positivity (more than 50%) was observed in four out of six cases (one of grade I, one grade II, and two grade III) (Figures 1H,K). Staining intensity was variable, from intermediate to strong, with higher intensity observed in more undifferentiated cells. Staining was generally cytoplasmic, and in one case, concurrent cytoplasmic and membranous staining was observed.

Three MNSTs were included, all of grade I. Of these, two expressed cytoplasmic CD117 with weak to strong intensity, with one being more than 50% positive (Figures 1I,L). The third case was negative.

### CD117 expression associations

When STSs were considered as a group, no significant association was found (positive/negative) between histological grade and CD117 expression (*p* = 0.574) (Table 4). With respect to stratifying STSs by different histotypes, all RMSs expressed CD117 and all leiomyosarcomas were CD117 negative.

**Table 4:** Association between CD117 expression and tumor grade in canine soft tissue sarcomas, evaluated using Pearson’s χ2 test.

	CD117 expression		
	Negative	Positive	*p*
All soft tissue sarcomas			
Grade I	20	11	0.574
Grade II	38	20	
Grade III	14	12	
Liposarcomas			
Grade I	16	2	0.012*
Grade II	21	2	
Grade III	2	3	
Perivascular wall tumors			
Grade I	2	8	0.190
Grade II	7	5	
Grade III	2	3	
Fibroblastic sarcomas			
Grade I	0	9	0.118
Grade II	1	3	

A *p*-value of <0.05 was considered statistically significant. * = *p* < 0.05.

In liposarcomas, a significant association was observed between histological grade and CD117 expression (positive/negative) (*p* = 0.012), and liposarcomas with higher tumor grades were more likely to show positive expression. No significant association was observed between CD117 expression and histological grade for PWTs (*p* = 0.190) and sarcomas of fibroblastic origin (*p* = 0.118) (Table 4). In addition, due to the low number of MNSTs, reliable statistical analysis could not be conducted.

Similarly, considering STSs as a group, no statistically significant association was identified (*p* = 0.417) between tumor grade and percentage of CD117-positive neoplastic cells.

While stratifying tumors by histotype, a significant association between tumor grade and percentage of CD117-positive neoplastic cells was observed in liposarcomas (*p* = 0.012), with higher-grade liposarcomas expressing CD117 in more than 75% of neoplastic cells. No association was observed between percentage of CD117-positive neoplastic cells and histological grade for PWTs (*p* = 0.150), sarcomas of fibroblastic origin (*p* = 0.057), and RMSs (*p* = 0.287) (Table 5).

**Table 5:** Association between the percentage of CD117-positive cells and tumor grade in canine soft tissue sarcomas, evaluated using Pearson’s χ2 test.

	Percentage of CD117-positive cells					
	<5%	5–25%	26–50%	51–75%	>75%	*p*
All soft tissue sarcomas						
Grade I	20	1	1	4	5	0.417
Grade II	38	6	1	3	10	
Grade III	14	0	1	3	8	
Liposarcomas						
Grade I	16	0	0	0	2	0.012*
Grade II	21	0	0	0	2	
Grade III	2	0	0	0	3	
Perivascular wall tumors						
Grade I	2	4	0	2	2	0.150
Grade II	7	0	1	1	3	
Grade III	2	0	0	2	1	
Fibroblastic sarcomas						
Grade I	0	0	1	1	7	0.057
Grade II	1	0	1	2	0	
Rhabdomyosarcomas						
Grade I	0	1	1	0	0	0.287
Grade II	0	0	1	0	0	
Grade III	0	1	0	2	0	

A *p*-value of <0.05 was considered statistically significant. * = *p* < 0.05.

### Mutations

Among the STSs included in the study, those with strong expression in more than 50% of the neoplastic cells, 22 cases were submitted for the assessment of mutations in exons 8, 9, and 11 of the c-kit gene: six sarcomas of fibroblastic origin (two fibrosarcomas and four myxosarcomas), six PWTs, three liposarcomas, five RMSs, and two MNSTs. No tumors had mutations in the tested exons of the c-kit gene.

## Discussion

CD117 expression and c-kit gene mutations were analyzed in different canine STS tumor types to explore the usefulness of targeted therapies in controlling the disease as STSs may often relapse, may be non-resectable, and can occasionally metastasize. Combining adjuvant target-specific anticancer therapies with current therapeutic protocols could be a useful approach as this could reduce the rate of local recurrence, extend the disease-free interval, and prevent or control nodal and distant metastases (54). This study highlights the importance of assessing potential molecular targets to expand therapeutic options for STSs, considering their frequent local recurrence and occasional metastatic behavior (7). In the present study, 37% of all analyzed canine STSs expressed CD117. CD117 expression was variable depending on histotypes, ranging from 100% expression in rhabdomyosarcomas to the lack of expression in leiomyosarcomas, and expression was variable depending by the histotype. In addition, studies have reported variations in CD117 expression in human STSs, from 0 to 20% of the cases (20, 55), and the lack of CD117 expression in leiomyosarcomas allows us to differentiate them from GISTs that are CD117 positive (56).

In this study, the highest percentage of CD117-positive cases was observed in RMSs. This observation is a novel finding that needs further confirmation as the cases included were few to draw strong conclusions. However, if this observation is confirmed in studies with a larger sample size, the use of TKIs in RMSs can be explored in clinical studies. This finding is of relevance as RMSs, especially alveolar and embryonic variants, may develop in young dogs (57). It is worth noting that most RMSs tend to be infiltrative and that some variants such as alveolar have been reported to develop distant metastases (57). RMSs in humans may also develop in pediatric patients and may express CD117 (15, 19, 55, 58). Furthermore, in human RMSs, CD117 expression correlates with the histological subtype (15), with a lack of CD117 expression in alveolar and botryoid RMSs and a consistent expression in RMSs with spindle cell morphology.

Although observations on RMSs are not always comparable between humans and animals and CD117 expression data in RMSs are lacking in veterinary medicine, the association between RMS subtype and CD117 expression in dogs should be investigated.

Tumors of fibroblastic origin expressed CD117 in 92% of cases (12 out of 13), with a case of fibrosarcoma being the only negative tumor. CD117 expression in tumors of fibroblastic origin has been previously analyzed only in canine oral fibrosarcomas (59), a tumor type that is consistently CD117 negative. Our findings are in contrast to those from reports on human STSs as sarcomas of fibroblastic origin have been found to express CD117 in percentages ranging from 0 to 74% of tumors (19, 55, 58).

Regarding both RMSs and tumors of fibroblastic origin, the present study lacks a sufficient number of cases to draw definitive conclusions about their CD117 expression, which necessitates future research. However, the preliminary results suggest that a majority of RMSs and tumors of fibroblastic origin may overexpress CD117, which indicates the opportunity for TKI-based therapy in dogs with unresectable disease with or without distant metastasis.

Two out of three canine MNSTs expressed strong cytoplasmic CD117, but the number of cases was too low to provide a representative percentage and conduct a reliable statistical analysis. Thus, this observation cannot be compared to, or supported by, other data in veterinary medicine. This limitation regarding the small number of MNSTs should be overcome in future studies, and the present results should be interpreted with caution. Nevertheless, our observation is in line with data reported in human medicine where up to 81% of MNSTs express CD117 (19, 55, 58).

In this study, 59% (16 of 27) of the PWTs variably expressed cytoplasmic CD117. PWTs are a common type of canine STSs developing more frequently in medium to large breed dogs and generally displaying a favorable prognosis (11). To our knowledge, a direct comparison is not possible due to the lack of similar studies on canine PWTs in veterinary medicine. However, in human medicine, the percentage of the formerly called hemangiopericytomas expressing CD117 varies from 0 to 20% (19). However, a proper comparison between canine and human PWTs is complex as human soft tissue hemangiopericytoma has been reclassified as solitary fibrous tumor (60); therefore, it is no longer included among PWTs. Other perivascular tumor types such as myopericytoma are rarer in humans than in dogs. In our cases, no association was found between CD117 expression and tumor grade, which is consistent with the finding on the expression of other TKRs in canine PWTs (61).

Of the liposarcomas included in this study, 15% (7 out of 46) expressed CD117; on the contrary, in human medicine, no CD117 expression has been found in liposarcomas (19, 55). This observation of CD117 expression in a minority of cases may support the hypothesis that CD117 overexpression is not primarily involved in cell proliferation and neoplastic transformation of canine liposarcomas (36).

Canine liposarcomas are rare but can be highly infiltrative and can recur locally (62), and the treatment of choice remains surgery. Among the STS histotypes included in the present study, liposarcoma was the only group of tumors where a significant association was observed between tumor grade and CD117 expression. No association has been previously reported between liposarcomas and CD117 expression in humans or in other animal species. However, a previous study has reported a correlation between tumor grade and CD117 expression in canine mast cell tumors (63). In addition, the association between the expression of platelet-derived growth factor receptor beta (PDGFRβ) and Ki67 labeling index has been reported in canine liposarcomas (36). Together, these findings suggest that multiple TKR-mediated pathways can be involved in the cellular proliferation of canine liposarcomas.

Although numerous liposarcomas were included in the present study, some variants were underrepresented. In future studies, to confirm the association of CD117 positivity with each morphological neoplastic variant, the number of cases needs to be increased. Attention should be paid to myxoid liposarcoma, which was uncommon in the present study but, based on recent studies, seems to represent a distinct entity compared with other types of canine liposarcomas (64, 65).

Consistent with previous reports in veterinary medicine (50), leiomyosarcomas included in the present study were always CD117 negative, a finding that further supports the role of CD117 as a diagnostic marker to differentiate leiomyosarcomas (CD117 negative) from GISTs (CD117 positive) (29) in dogs and in some human GIST cases (56).

This observation is in contrast to those from studies on human leiomyosarcomas, which have been reported to express CD117 in 37–82% of cases (18, 20, 58), whereas other studies have reported CD117-negative results for leiomyosarcomas (55, 66).

Notably, a high interstudy variability regarding CD117 expression in human sarcomas was observed, which constrains a more accurate comparison between canine and human STSs and CD117 expression. In human medicine literature, this variability has been attributed to the application of different classification systems, possible misclassifications of some tumors, and technical inconsistencies in the IHC protocols applied in different studies (55).

Regarding c-kit mutations, exons 8, 9, and 11 were chosen because their mutations have been more frequently reported in dogs (67, 68). In the present study, none of the 22 canine STSs assessed showed mutations in these exons. These same exons have been demonstrated to frequently contain activating mutations in canine MCTs, leading to proliferation independent of the action of the growth factor (67). The mutational state of the c-kit gene can influence the efficacy of TKIs in both humans (21, 69) and dogs (70, 71). In human medicine, tumors with mutations in exon 11 appear to be more responsive to TKIs than those with mutations in exon 9, and tumors without mutations seem not to benefit from this therapeutic approach (19, 72).

Similarly, dogs having MCTs with an internal tandem duplication (ITD) mutation in c-kit gene exon 11 seem approximately two times as responsive to treatment with the TKI toceranib phosphate as dogs with MCTs harboring the wild-type c-kit gene (24, 25, 27, 43).

In canine MCTs, a correlation between tumor grade and the presence of mutations in the juxtamembrane domain of CD117 has been reported, with mutations more frequently observed in grade II and III MCTs (73). Furthermore, MCTs with ITD mutations have a significantly higher proliferation index, as measured by Ki67 IHC staining, and a significantly higher cell cycle progression rate, as measured by AgNOR histochemical staining, compared with MCTs without an ITD mutation (26).

In canine GISTs, mutations in c-kit exon 11 are involved in tumor emergence by inducing ligand-independent phosphorylation, which suggests the therapeutic potential of TKIs in this tumor type (29, 39). Nevertheless, the efficacy of TKIs has been reported in some cases of dogs affected by GISTs showing strong IHC cytoplasmic positivity for CD117 and lacking mutations in c-kit exons 8 and 11 (39). This is a relevant finding as the lack of c-kit mutations in a tumor does not imply a lack of responsiveness to TKI treatment as long as CD117 is expressed, which suggests the involvement of alternative mechanisms in CD117 activation and response to TKI treatments. These alternative mechanisms can include overexpression of non-mutated CD117, intervention of other RTKs, mutated or overactive effector molecules in CD117 downstream pathways, or mutations in unevaluated exons.

Because alternative mechanisms may drive tumor growth, alongside or independent of c-kit mutations, a more in-depth investigation into these mechanisms is recommended to support the use of TKIs alongside agents targeting putative activating mechanisms (e.g., inhibition of the downstream pathway).

While only three c-kit exons 8, 9 and 11 were assessed for mutations in the present study, it is speculated that other exons also contribute to oncogenesis in canine malignancies. Mutations in exons 12, 14, and 17 have been reported in canine MCTs (51, 67, 70). In humans, mutations in c-kit exon 13 have been observed in GISTs (66) and mutations in exon 17 have been reported in mastocytosis (21, 74). Thus, oncogenesis in canine sarcomas could be driven by mutations in exons other than those investigated in the present study.

This study provides novel insights into CD117 expression in different histotypes of canine STSs but has some limitations, including underrepresentation of some histotypes, low statistical reliability of the analyses conducted, a lack of information regarding the mutational status of exons 12, 13 and 17, and a lack of a more objective quantification of CD117 immunolabeling using image analysis. Another limitation is the lack of follow-up data regarding the cases included, which prevents us from drawing conclusions about the correlation between CD117 expression and clinical outcome, with or without TKI therapy. Further prospective studies are needed to address these knowledge gaps.

## Conclusion

To the best of the authors’ knowledge, the present study is the first investigative study of CD117 expression and c-kit gene mutation in different canine STS histotypes. Our findings suggest that analyzing CD117 expression may be useful in several canine STS tumor types, especially in those exhibiting clinically aggressive behavior and/or high tumor grade, to include additional adjuvant treatment protocols. While this study did not clearly demonstrate the role of mutations in c-kit exons 8, 9, and 11 in STSs, a more extensive assessment of c-kit gene mutations and their therapeutic and prognostic significance in canine STSs should be conducted in future studies.

## Data Availability

The raw data supporting the conclusions of this article will be made available by the authors, without undue reservation.
