# Supplementary material for: CD117 (KIT) in canine soft tissue sarcoma: an immunohistochemical and c-kit gene mutation assessment
Source: Front Vet Sci. 2025 Apr 9;12:1572923. doi: 10.3389/fvets.2025.1572923 (PMC12014545; doi:10.3389/fvets.2025.1572923)
Supplement: Supplementary file 1 [file Table_1.docx]

**Supplementary Table 1.** Signalment and clinical details of Soft Tissue Sarcoma cases included in the study.

| **Case N.** | **Breed** | **Sex** | **Age** | **Histotype** | **Localization** | **Differen-**  **tiation score** | **Mitotic count** | **Necrosis** | **Grade** | **CD117 positivity** | **CD 117 intensity** | **CD 117 localization** |
| --- | --- | --- | --- | --- | --- | --- | --- | --- | --- | --- | --- | --- |
| **1** | German Sheperd | M | 11 | Liposarcoma (pleomorphic) | NA | 3 | 7 | ≤50% | 2 | - |  |  |
| **2** | Golden Retriever | M | 13 | Liposarcoma (pleomorphic) | Hindlimb | 2 | 2 | Absent | 1 | - |  |  |
| **3** | NA | M | 7 | Liposarcoma (pleomorphic) | Trunk | 3 | 2 | ≤50% | 2 | - |  |  |
| **4** | M.Shepherd | M | 14 | Liposarcoma (well differentiated) | Inguinal | 2 | 1 | ≤50% | 2 | - |  |  |
| **5** | Labrador | M | 13 | Liposarcoma (pleomorphic) | Forelimb | 3 | 7 | ≤50% | 2 | - |  |  |
| **6** | Poodle | M | 14 | Liposarcoma (pleomorphic) | Axilla | 2 | 5 | Absent | 1 | - |  |  |
| **7** | NA | M | 4 | Liposarcoma (pleomorphic) | Hindlimb | 3 | 8 | ≤50% | 2 | - |  |  |
| **8** | Crossbreed | M | 8 | Liposarcoma (pleomorphic) | Neck | 2 | 1 | Absent | 1 | - |  |  |
| **9** | German Sheperd | M | 8 | Liposarcoma (well differentiated) | Trunk | 2 | 1 | ≤50% | 2 | - |  |  |
| **10** | Rottweiler | M | 12 | Liposarcoma (well differentiated) | Inguinal | 1 | 1 | ≤50% | 1 | ++++ | Intermediate | Cytoplasm |
| **11** | M.Shepherd | M | 10 | Liposarcoma (pleomorphic) | Axilla | 3 | 8 | ≤50% | 2 | - |  |  |
| **12** | Crossbreed | M | 13 | Liposarcoma (well differentiated) | Trunk | 1 | 1 | ≤50% | 1 | - |  |  |
| **13** | Hound | F | 4 | Liposarcoma (well differentiated) | Trunk | 2 | 2 | Absent | 2 | - |  |  |
| **14** | Labrador | M | 11 | Liposarcoma (pleomorphic) | Hindlimb | 2 | 2 | Absent | 2 | - |  |  |
| **15** | Labrador | M | 10 | Liposarcoma (pleomorphic) | Axilla | 2 | 4 | Absent | 1 | - |  |  |
| **16** | Labrador | M | 10 | Liposarcoma (well differentiated) | Hindlimb | 1 | 7 | Absent | 1 | - |  |  |
| **17** | Bassetthound | M | 9 | Liposarcoma (dedifferentiated) | Spinal | 3 | 56 | Absent | 3 | - |  |  |
| **18** | Labrador | M | 10 | Liposarcoma (pleomorphic) | Neck | 2 | 2 | ≤50% | 2 | - |  |  |
| **19** | Pointer | M | 10 | Liposarcoma (pleomorphic) | Trunk | 2 | 2 | ≤50% | 2 | - |  |  |
| **20** | Crossbreed | M | 13 | Liposarcoma (pleomorphic) | Perineum | 1 | 3 | Absent | 1 | - |  |  |
| **21** | Crossbreed | F | 12 | Liposarcoma (well differentiated) | Hindlimb | 2 | 6 | Absent | 1 | - |  |  |
| **22** | Crossbreed | F | 6 | Liposarcoma (dedifferentiated) | Trunk | 3 | 17 | Absent | 2 | ++++ | Strong | Cytoplasm |
| **23** | Crossbreed | M | 7 | Liposarcoma (pleomorphic) | Trunk | 2 | 6 | ≤50% | 2 | - |  |  |
| **24** | Crossbreed | M | 9 | Liposarcoma (pleomorphic) | Trunk | 3 | 3 | ≤50% | 2 | - |  |  |
| **25** | Fox Terrier | M | 12 | Liposarcoma (pleomorphic) | NA | 3 | 6 | ≤50% | 2 | - |  |  |
| **26** | Labrador | MC | 13 | Liposarcoma (well differentiated) | Perineum | 2 | 7 | Absent | 1 | - |  |  |
| **27** | Bassetthound | M | 10 | Liposarcoma (well differentiated) | Axilla | 1 | 4 | Absent | 1 | - |  |  |
| **28** | Golden Retriever | M | 9 | Liposarcoma (pleomorphic) | Hindlimb | 2 | 5 | ≤50% | 2 | - |  |  |
| **29** | Labrador | M | 11 | Liposarcoma (pleomorphic) | Forelimb | 3 | 18 | ≤50% | 3 | ++++ | Strong | Cytoplasm |
| **30** | Labrador | M | 10 | Liposarcoma (dedifferentiated) | Hindlimb | 3 | 29 | Absent | 3 | - |  |  |
| **31** | Labrador | M | 10 | Liposarcoma (pleomorphic) | Inguinal | 1 | 2 | ≤50% | 1 | - |  |  |
| **32** | Golden Retriever | F | 13 | Liposarcoma (well differentiated) | Axilla | 1 | 2 | Absent | 1 | - |  |  |
| **33** | Beagle | MC | 13 | Liposarcoma (pleomorphic) | Trunk | 2 | 8 | ≤50% | 2 | - |  |  |
| **34** | Crossbreed | F | 14 | Liposarcoma (well differentiated) | Axilla | 1 | 2 | ≤50% | 1 | - |  |  |
| **35** | Fox Terrier | M | 10 | Liposarcoma (pleomorphic) | Trunk | 2 | 3 | Absent | 1 | - |  |  |
| **36** | Crossbreed | F | 10 | Liposarcoma (well differentiated) | Trunk | 1 | 1 | Absent | 1 | - |  |  |
| **37** | Labrador | M | 10 | Liposarcoma (well differentiated) | Neck | 2 | 4 | ≤50% | 2 | - |  |  |
| **38** | NA | F | 10 | Liposarcoma (pleomorphic) | Trunk | 3 | 6 | Absent | 2 | ++++ | Intermediate | Cytoplasm |
| **39** | Beagle | MC | NA | Liposarcoma (well differentiated) | Trunk | 1 | 3 | ≤50% | 1 | - |  |  |
| **40** | Crossbreed | M | 13 | Liposarcoma (pleomorphic) | Perineum | 3 | 11 | Absent | 2 | - |  |  |
| **41** | Labrador | F | 15 | Liposarcoma (myxoid) | Trunk | 3 | 3 | >50% | 3 | ++++ | Strong | Cytoplasm |
| **42** | Great Dane | NA | 8 | Liposarcoma (myxoid) | Tail | 3 | 13 | Absent | 2 | - |  |  |
| **43** | Rottweiler | M | NA | Liposarcoma (pleomorphic) | Axilla | 2 | 4 | ≤50% | 2 | - |  |  |
| **44** | Crossbreed | FN | 11 | Liposarcoma (myxoid) | Forelimb | 2 | 10 | ≤50% | 2 | - |  |  |
| **45** | Crossbreed | F | 16 | Liposarcoma (myxoid) | Axilla | 2 | 35 | ≤50% | 3 | ++++ | Strong | Cytoplasm |
| **46** | German Spitz | M | 8 | liposarcoma (well differentiated) | Hindlimb | 1 | 5 | Absent | 1 | ++++ | Strong | Cytoplasm |
| **47** | Siberian Husky | M | 11 | Perivascular wall tumor | Trunk | 3 | 3 | ≤50% | 2 | ++++ | Intermediate | Cytoplasm |
| **48** | Labrador | M | 13 | Perivascular wall tumor | Hindlimb | 2 | 9 | Absent | 1 | + | Intermediate | Nucleus |
| **49** | Crossbreed | MC | 13 | Perivascular wall tumor | Hindlimb | 1 | 3 | Absent | 1 | +/- | Weak | Cytoplasm |
| **50** | Crossbreed | FN | 13 | Perivascular wall tumor | NA | 1 | 7 | Absent | 1 | ++++ | Intermediate | Cytoplasm |
| **51** | German Sheperd | MC | 10 | Perivascular wall tumor | Hindlimb | 1 | 8 | Absent | 1 | + | Intermediate | Cytoplasm |
| **52** | Labrador | M | 12 | Perivascular wall tumor | Forelimb | 2 | 0 | Absent | 1 | + | Strong | Cytoplasm |
| **53** | Crossbreed | FN | 8 | Perivascular wall tumor | Forelimb | 1 | 4 | Absent | 1 | +++ | Intermediate | Cytoplasm |
| **54** | Crossbreed | M | 11 | Perivascular wall tumor | Hindlimb | 1 | 12 | Absent | 1 | +++ | Strong | Cytoplasm |
| **55** | Labrador | M | 10 | Perivascular wall tumor | Forelimb | 2 | 8 | Absent | 1 | ++++ | Strong | Cytoplasm |
| **56** | Poodle | M | 9 | Perivascular wall tumor | Hindlimb | 2 | 5 | Absent | 1 | + | Intermediate | Cytoplasm |
| **57** | Siberian Husky | FN | 13 | Perivascular wall tumor | Hindlimb | 1 | 17 | Absent | 1 | - |  |  |
| **58** | Crossbreed | M | 9 | Perivascular wall tumor | Trunk | 2 | 10 | Absent | 2 | - |  |  |
| **59** | Boxer | MC | 12 | Perivascular wall tumor | Forelimb | 1 | 13 | ≤50% | 2 | +/- | Weak | Cytoplasm |
| **60** | Maltese | F | 8 | Perivascular wall tumor | Neck | 1 | 10 | ≤50% | 2 | - |  |  |
| **61** | Rottweiler | F | 11 | Perivascular wall tumor | Forelimb | 2 | 6 | ≤50% | 2 | ++++ | Strong | Cytoplasm |
| **62** | Boxer | FN | 8 | Perivascular wall tumor | Hindlimb | 2 | 10 | ≤50% | 2 | ++++ | Intermediate | Cytoplasm |
| **63** | Pomeranian | M | 11 | Perivascular wall tumor | Hindlimb | 1 | 28 | Absent | 2 | ++ | Weak | Cytoplasm |
| **64** | Jack russel | M | 12 | Perivascular wall tumor | Forelimb | 1 | 10 | 0 | 2 | +++ | Weak | Cytoplasm |
| **65** | Crossbreed | MC | 13 | Perivascular wall tumor | Trunk | 2 | 13 | ≤50% | 2 | - |  |  |
| **66** | Shar pei | F | 11 | Perivascular wall tumor | Trunk | 2 | 17 | ≤50% | 2 | +/- | Weak | Cytoplasm |
| **67** | Crossbreed | MC | 5 | Perivascular wall tumor | Inguinal | 2 | 20 | Absent | 2 | +/- | Weak | Cytoplasm |
| **68** | Crossbreed | F | 11 | Perivascular wall tumor | Hindlimb | 2 | 10 | Absent | 2 | - |  |  |
| **69** | Crossbreed | M | 14 | Perivascular wall tumor | Trunk | 3 | 42 | >50% | 3 | ++++ | Intermediate to Strong | Cytoplasm |
| **70** | Bassetthound | M | 2 | Perivascular wall tumor | Trunk | 2 | 53 | Absent | 3 | - |  |  |
| **71** | Crossbreed | M | 12 | Perivascular wall tumor | Forelimb | 2 | 31 | ≤50% | 3 | - |  |  |
| **72** | Bassetthound | FN | 15 | Perivascular wall tumor | Forelimb | 2 | 25 | ≤50% | 3 | +++ | Weak | Cytoplasm |
| **73** | Crossbred | M | 13 | Perivascular wall tumor | Trunk | 2 | 35 | ≤50% | 3 | +++ | Intermediate | Cytoplasm |
| **74** | Schnauzer nano | FN | 10 | Leiomyosarcoma | Retroperitoneum | 1 | 1 | Absent | 1 | - |  |  |
| **75** | Hound | FN | 8 | Leiomyosarcoma | Forelimb | 2 | 5 | Absent | 1 | - |  |  |
| **76** | Crossbreed | M | 9 | Leiomyosarcoma | Hindlimb | 2 | 2 | Absent | 1 | - |  |  |
| **77** | Beagle | M | 10 | Leiomyosarcoma | Perineum | 2 | 37 | >50% | 3 | - |  |  |
| **78** | Labrador | MC | 5 | Leiomyosarcoma | Perineum | 3 | 23 | ≤50% | 3 | - |  |  |
| **79** | Crossbreed | M | 12 | Leiomyosarcoma | Hindlimb | 3 | 1 | ≤50% | 2 | - |  |  |
| **80** | Crossbreed | F | 12 | Leiomyosarcoma | Pelvis | 1 | 1 | ≤50% | 1 | - |  |  |
| **81** | Crossbreed | F | 10 | Leiomyosarcoma | Perineum | 2 | 10 | ≤50% | 2 | - |  |  |
| **82** | Crossbreed | F | 9 | Leiomyosarcoma | Retroperitoneum | 2 | 2 | ≤50% | 2 | - |  |  |
| **83** | Doberman Pinscher | F | 4 | Leiomyosarcoma | Pelvis | 2 | 6 | Absent | 1 | - |  |  |
| **84** | Golden Retriever | F | 11 | Leiomyosarcoma | Perineum | 1 | 9 | ≤50% | 1 | - |  |  |
| **85** | Crossbreed | FN | 12 | Leiomyosarcoma | Forelimb | 2 | 9 | Absent | 1 | - |  |  |
| **86** | Labrador | FN | 10 | Leiomyosarcoma | Pelvis | 2 | 3 | ≤50% | 2 | - |  |  |
| **87** | Crossbreed | M | 4 | Leiomyosarcoma | Mesentery | 2 | 8 | ≤50% | 2 | - |  |  |
| **88** | Crossbreed | M | 11 | Leiomyosarcoma | Perineum | 1 | 2 | Absent | 1 | - |  |  |
| **89** | Crossbreed | M | 14 | Leiomyosarcoma | Retroperitoneum | 1 | 7 | Absent | 1 | - |  |  |
| **90** | Golden Retriever | M | 9 | Leiomyosarcoma | Forelimb | 1 | 2 | Absent | 1 | - |  |  |
| **91** | Crossbreed | M | 15 | Leiomyosarcoma | Retroperitoneum | 1 | 1 | Absent | 1 | - |  |  |
| **92** | Crossbreed | M | 15 | Leiomyosarcoma | Lip | 2 | 83 | ≤50% | 3 | - |  |  |
| **93** | Golden Retriever | FN | 12 | Leiomyosarcoma | Hindlimb | 2 | 16 | Absent | 2 | - |  |  |
| **94** | Kurzhaar | F | 6 | Fibrosarcoma | Trunk | 2 | 1 | ≤50% | 2 | +++ | Intermediate | Cytoplasm |
| **95** | Crossbreed | FN | 9 | Fibrosarcoma | Oral cavity | 2 | 3 | Absent | 1 | ++ | Intermediate | Cytoplasm |
| **96** | German Sheperd | F | 7 | Fibrosarcoma | Nose | 2 | 0 | Absent | 1 | ++++ | Strong | Cytoplasm |
| **97** | Dobermann | FN | 7 | Fibrosarcoma | NA | 3 | 6 | ≤50% | 2 | +++ | Intermediate to Strong | Cytoplasm |
| **98** | Pointer | F | 11 | Fibrosarcoma | Hindlimb | 2 | 0 | Absent | 1 | ++++ | Strong | Cytoplasm |
| **99** | Crossbreed | M | 9 | Fibrosarcoma | Lip | 3 | 2 | Absent | 2 | ++ | Strong | Cytoplasm |
| **100** | Akita Inu | FN | 8 | Fibrosarcoma | Neck | 3 | 0 | >50% | 2 | - |  |  |
| **101** | Crossbreed | NA | NA | Myxosarcoma | Trunk | 1 | 0 | Absent | 1 | ++++ | Strong | Cytoplasm |
| **102** | Crossbreed | F | 5 | Myxosarcoma | NA | 2 | 3 | Absent | 1 | ++++ | Strong | Cytoplasm |
| **103** | Standard Schnauzer | M | 1 | Myxosarcoma | Forelimb | 1 | 2 | Absent | 1 | ++++ | Strong | Cytoplasm |
| **104** | Beagle | F | 16 | Myxosarcoma | Hindlimb | 3 | 1 | Absent | 1 | +++ | Intermediate | Cytoplasm |
| **105** | Rottweiler | F | 7 | Myxosarcoma | Eyelid | 2 | 0 | Absent | 1 | ++++ | Intermediate to Strong | Cytoplasm |
| **106** | Crossbreed | FN | 10 | Myxosarcoma | Forelimb | 2 | 0 | Absent | 1 | ++++ | Strong | Cytoplasm |
| **107** | English Setter | F | 1 | Rhabdomyosarcoma (pleomorphic) | Nasal Cavity | 3 | 29 | Absent | 3 | +++ (fusate cells)  ++++ (pleomorphic cells) | Intermediate (fusate cells) to Strong (pleomorphic cells) | Cytoplasm |
| **108** | NA | NA | NA | Rhabdomyosarcoma (pleomorphic) | NA | 2 | 3 | Absent | 1 | + (indifferentiated cells) | Intermediate | Cytoplasm |
| **109** | Coton de Tulear | M | 10 | Rhabdomyosarcoma | Axilla | 3 | 2 | ≤50% | 2 | +++ | Weak to Strong | Cytoplasm |
| **110** | NA | F | 11 | Rhabdomyosarcoma | Forelimb | 2 | 23 | <50% | 3 | + | Intermediate | Cytoplasm, Membrane |
| **111** | Labrador | F | 4 | Rhabdomyosarcoma (embrionario+pleomorphic) | Head (masseter) | 1 | 2 | Absent | 1 | +++ | Strong | Cytoplasm |
| **112** | German Sheperd | M | 1 | Rhabdomyosarcoma | Head | 3 | 63 | >50% | 3 | ++++ | Strong | Cytoplasm |
| **113** | Crossbreed | FN | 9 | NST | Trunk | 2 | 5 | Absent | 1 | + | Weak | Cytoplasm |
| **114** | Golden Retriever | M | 10 | NST | Forelimb | 2 | 9 | Absent | 1 | ++++ | Strong | Cytoplasm |
| **115** | Crossbreed | FN | 8 | NST | Axilla | 2 | 3 | Absent | 1 | - |  |  |

Symbols: -, negative; -/+, <5% neoplastic cells; +, 5-25% neoplastic cells; ++, 26-50% neoplastic cells; +++, 51-75% neoplastic cells; ++++, >75% neoplastic cells; M, male; MC, castrated male; F, female; FN, neutered female; NA, Not Available; PWT, perivascular wall tumor; NST, nerve sheath tumor.
